# Calpain-Mediated Mitochondrial Damage: An Emerging Mechanism Contributing to Cardiac Disease

**DOI:** 10.3390/cells10082024

**Published:** 2021-08-08

**Authors:** Mengxiao Zhang, Grace Wang, Tianqing Peng

**Affiliations:** 1 Institutes of Biology and Medical Sciences, Soochow University, Suzhou 215123, China; mzhan628@uwo.ca; 2 School of Pharmacy, Bengbu Medical College, Bengbu 233000, China; 3 Department of Pathology and Laboratory Medicine, Western University, London, ON N6A 5C1, Canada; 4 Department of Medicine, University of Toronto, Toronto, ON M5S 1A1, Canada; Graceyang.wang@mail.utronto.ca; 5 Department of Medicine, Western University, London, ON N6A 5C1, Canada; 6 Lawson Health Research Institute of London Health Sciences Centre, London, ON N6A 5W9, Canada

**Keywords:** calpain, mitochondria, cardiac disease

## Abstract

Calpains belong to the family of calcium-dependent cysteine proteases expressed ubiquitously in mammals and many other organisms. Activation of calpain is observed in diseased hearts and is implicated in cardiac cell death, hypertrophy, fibrosis, and inflammation. However, the underlying mechanisms remain incompletely understood. Recent studies have revealed that calpains target and impair mitochondria in cardiac disease. The objective of this review is to discuss the role of calpains in mediating mitochondrial damage and the underlying mechanisms, and to evaluate whether targeted inhibition of mitochondrial calpain is a potential strategy in treating cardiac disease. We expect to describe the wealth of new evidence surrounding calpain-mediated mitochondrial damage to facilitate future mechanistic studies and therapy development for cardiac disease.

## 1. Introduction

Cardiovascular disease (CVD) remains the leading cause of death worldwide. According to the World Health Organization, approximately 17.9 million people died from CVDs in 2016, accounting for 31% of all global deaths [1]. Mitochondrial dysfunction represents an important mechanism underlying cardiac disease. Mitochondria are one of the most important organelles in living organisms. They are not only the center of cell energy metabolism, but are also critical to a wide range of physiological conditions, including intracellular iron-sulfur cluster formation, calcium homeostasis, apoptosis and amino acid metabolism [2,3]. Well-functioning mitochondria are essential for maintaining cardiac function. Indeed, the majority of the energy in the form of ATP consumed by the heart (up to 95%) is produced by the mitochondria [4], organelles accounting for about 30%-40% volume of cardiomyocytes [5]. Mitochondrial dysfunction has been widely observed in diseased hearts, and directly contributes to myocardial dysfunction through energy depletion. Dysfunctional mitochondria generate excessive reactive oxygen species (ROS), which surpasses the capacity of the antioxidant system to scavenge ROS, thereby causing cardiomyocyte death [6,7]. This loss of cardiomyocytes is a fundamental process leading to adverse cardiac remodeling and heart failure, as the regeneration of cardiomyocytes is extremely limited in the heart [8]. Excessive ROS production in mitochondria may also trigger an inflammatory response, leading to further tissue damage and myocardial remodeling [9]. While many studies have identified the role of mitochondrial dysfunction in cardiac diseases, the underlying mechanisms remain incompletely understood.

Accumulating evidence has suggested a pivotal role of the calpain system in mediating mitochondrial dysfunction in diseased hearts [10,11]. Calpains are a family of calcium-dependent neutral cysteine proteases that cleave a broad spectrum of substrates upon activation [12]. Calpains are involved in various physiological and pathological processes, including, but not limited to, cell proliferation [13], cell migration [14], cytoskeletal degradation [15], apoptosis [16], autophagy [17], and inflammation [18]. Calpain is indispensable for embryonic development and protects the heart from hemodynamic stress [19,20]. Moreover, the work from Zheng et al. has unveiled an important role of calpain in mediating the hypoxia-inducible factor-1α (HIF-1α) signaling pathway [21]. HIF-1α is a transcription factor that plays critical roles in cellular adaptive responses to hypoxia, and is one of the first adaptations of human myocardium to ischemia or infarction [22]. Upregulated HIF-1α promotes angiogenesis and benefits tissue perfusion in animal models of ischemic cardiovascular disease [23]. The nuclear localization of HIF-1α requires the facilitation of filamin A (FLNA) fragments. Hypoxia induces a calpain-dependent cleavage of FLNA, generating a C-terminal fragment that translocates to the nucleus, where it facilitates the recruitment of HIF-1α to target gene promoters and enhances HIF-1α function [21]. This is in line with the notion that functional calpain may play important roles in angiogenesis [24,25]. A dysfunctional calpain system, however, contributes to cardiac injury under various pathological conditions including diabetes [26,27], myocardial infarction [28], ischemia/reperfusion injury [29], sepsis [30], viral myocarditis [18], and heart failure [12]. Overactivation of calpain leads to the disruption of cellular proteins responsible for maintaining sarcomere structure [31] and cell contractility [32], which directly compromises cardiac pump function. Calpain directly degrades myosin light chain kinase in a time- and dose-dependent manner, thus contributing to pressure-overload-induced cardiac hypertrophy [33]. Recent work from Song’s group unveiled the pivotal role of calpain in cleaving juncphilin-2, a structural protein required for cardiac excitation–contraction (E–C) coupling, leading to disruption of E–C coupling and driving heart failure progression [12,34]. A recent study also showed that calpain-2 cleaves junctophilin-2 in heart failure and that the C-terminal fragment of junctophilin-2 generated by calpain-2 translocates to the cardiomyocyte nucleus, which may promote isoproterenol-induced hypertrophy in cardiomyocytes [35]. Our previous works on various mouse models of cardiac diseases have demonstrated that activation of cardiac calpain impairs ATP synthase activity [36,37], induces massive ROS production [38], elicits endoplasmic reticulum stress [39] and promotes cell death [40]. Moreover, suppression of calpain with pharmacological inhibitors or genetic approaches has shown promise in alleviating cardiac injury and dysfunction related to diabetes [41,42], ischemia/reperfusion [10,43], sepsis [37,44], pressure overload [12], and drug-induced myocardial damage [45,46], all of which support the critical role of calpain in cardiac diseases. Over-expression of calpastatin, an endogenous inhibitor of calpain, reduces cardiac hypertrophy induced by angiotensin-II in transgenic mice [47]. These studies also suggest that calpain activation may contribute to the progression of heart failure, a notion further strengthened by the evidence that cardiac over-expression of calpain-1 is sufficient to cause heart failure in calpain-1 transgenic mice [40]. Taken together, calpain inhibition may represent a promising therapeutic strategy for cardiac diseases and heart failure treatment. In this review, we discuss the role of calpain in mediating mitochondrial damage and its underlying mechanisms, and also evaluate whether targeted inhibition of mitochondria-localized calpain (mito-calpain) is a potential strategy to treat cardiac diseases.

## 2. Regulation of Calpain System

Thus far, 15 calpain isoforms have been identified in mammals, which can be divided into conventional calpains and non-conventional calpains [48]. Conventional calpains are comprised of two isoforms: calpain-1 (μ-calpain), which requires micromolar Ca^2+^ for activation, and calpain-2 (m-calpain), which requires millimolar Ca^2+^ for activation. These two isoforms, as well as calpain-10, a mitochondrial subtype, are ubiquitously expressed across all tissues [49,50]. Unconventional calpains are often expressed in a tissue-specific manner. For instance, calpain-3 is found in skeletal muscle and submandibular glands [51], while calpain-8 and 9 are predominantly expressed in the gastrointestinal tract [52]. In this review, the term calpain(s) will refer to conventional calpains (calpain-1 and calpain-2) unless otherwise stated.

Both calpain-1 and calpain-2 are heterodimers composed of an 80 kDa catalytic subunit and a 28 kDa regulatory subunit. The catalytic subunit of calpain-1 and calpain-2, namely CAPN1 and CAPN2, are encoded by *Capn1* and *Capn2*, respectively. The detailed structure of calpains are well described in previous reviews [25,53]. The regulatory subunit, namely CAPNS1 (or CAPN4), is common to conventional calpains and encoded by *Capns1* (or *Capn4*). CAPNS1 is essential for the stability and activity of conventional calpains, since deletion of the *Capns1* gene completely abolishes the activities of calpain-1 and calpain-2 [39,54].

Calpain activity is tightly regulated by intracellular Ca^2+^ concentration and an endogenous calpain inhibitor, calpastatin, which specifically inhibits calpain-1 and -2 without effecting the activity of other proteases [19]. Calpastatin is specifically activated by active calpain through limited and Ca^2+^-dependent proteolysis. Calpastatin binds to calpain in a ratio of up to 1:4 [55] and thereby renders calpain inactive [56]. In certain pathological conditions such as ischemic myocardium, the expression of calpain increases, while the expression of calpastatin remains unchanged, leading to an imbalance in the calpain system and subsequent calpain activation [57]. Moreover, Aminah A. Loonat and colleagues have recently identified calpastatin as a substrate of p38γ in the heart, and p38γ-mediated phosphorylation of calpastatin dampens its inhibitory effect on calpain [58], indicating that calpastatin modification also serves as a mechanism of calpain activation in the heart. In conditions characterized by Ca^2+^ mishandling, both *Capns1* deletion and calpastatin overexpression successively inhibit calpain activity and prevent detrimental effects elicited by calpain over-activation [37]. Although calpain-1 and calpain-2 usually require Ca^2+^ for activation, studies also suggest that extracellular signal-regulated kinase (ERK)-mediated phosphorylation may exert a similar activation as Ca^2+^ or lower the Ca^2+^ concentration required for calpain-2 activation [59,60]. In addition, phosphatidylinositol 4,5-bisphosphate was reported to regulate calpain-2 activation [61]. Nevertheless, the regulatory mechanisms of calpain activation warrant further investigation.

Calpain is subject to spatial regulation and translocates among various subcellular compartments under stressed conditions. Following an increase in intracellular Ca^2+^, calpastatin dissociates from calpains to allow their membrane translocation [62]. Once associated with the membrane, calpains undergo autoproteolysis to generate the fully active enzyme [62]. Cell membrane recruitment of calpain is indicative of calpain activation and positively correlates with myocardial injury [29,63]. In isolated rat hearts, ischemia induces translocation of calpain-2 from cytosol to the sarcolemma membrane, where calpain activation occurs during the reperfusion period [64]. Similarly, in rat hearts subjected to calcium paradox, both calpain-1 and calpain-2 translocate to the sarcolemma membrane for activation, leading to cleavage of α-fodrin and impairment of membrane integrity, thus contributing to myocardial dysfunction [65]. Nuclear translocation of calpain-2 was reported in cardiomyocytes of tail-suspended rats and transverse aortic constriction rats, and associated with apoptosis in cardiomyocytes [66,67]. Notably, our previous studies in mouse models of endotoxemia and diabetes demonstrated that both lipopolysaccharides (LPS) and diabetic conditions induced translocation of calpain-1 to mitochondria in cardiomyocytes, which elicits excessive mitochondrial ROS production and myocardial dysfunction [36,37]. We further reported that hypoxia/reoxygenation and global ischemia/reperfusion increased calpain-1 in the mitochondria of cultured cardiomyocytes and isolated whole hearts, respectively [42]. Similarly, other studies have demonstrated an increase in calpain activity in the mitochondria of ischemic heart [68,69]. These findings have implicated mito-calpain in cardiac pathology. In mammal hearts, three calpain isoforms have been identified in the mitochondria, namely calpain-1, calpain-2 and calpain-10 [49,69]. The N-terminus of calpain-1 and calpain-10 contain a similar amphipathic structure, which is considered a mitochondrial target sequence, a potential mechanism for translocation of calpain to mitochondria [70]. In mouse hearts, the large subunit of mito-calpain-1 is found in the crude mitochondrial outer membrane, intermembrane space, and matrix [49]. In rat hearts, calpain-2 has been detected in mitochondria and localized to the mitochondrial matrix [69]. Calpain-10 is considered a mitochondrial subtype and is detected in every component of mitochondria [50]. The basic structure and calcium dependency of mito-calpain are similar to those of cytosolic calpain (cyto-calpain) [71], but there are minor differences in inhibitor susceptibility and optimum working pH between mito-calpain and cyto-calpain [71]. More in-depth works are needed to elucidate the biochemical and functional characters of mito-calpain and cyto-calpain in the heart.

## 3. Role of Calpain in Mediating Mitochondrial Damage during Cardiac Disease

Calpain contributes to various cardiac diseases, as calpain activation and myocardial injury often occur in a temporal and spatial sequence [72], and calpain inhibition has been shown to be an important strategy of cardioprotection [28,73]. Cyto-calpain and mito-calpain work separately yet synergistically in mediating mitochondrial damage under pathological conditions. This chapter discusses cyto-calpain- and mito-calpain-mediated mitochondrial damage in cardiac pathology and their potential mechanisms.

### 3.1. Cyto-Calpain-Mediated Mitochondrial Damage

**Figure 1:**
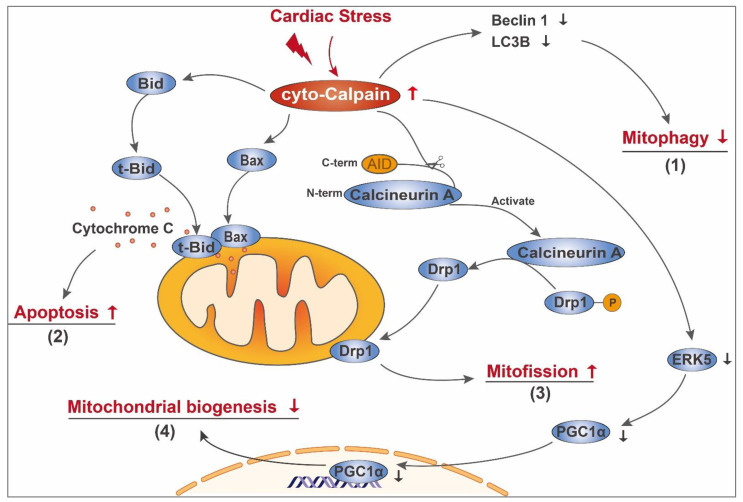
Schematic diagram of cyto-calpain-mediated mitochondrial damage in the diseased heart. In the diseased heart, cardiac stress leads to cyto-calpain activation. Activated cyto-calpain (**1**) decreases the content of Beclin 1 and LC3B, thus impairing mitophagy; (**2**) cleaves Bid and Bax, facilitating their mitochondrial translocation and subsequent leakage of pro-apoptotic components, such as cytochrome C; (**3**) activates calcineurin A by direct removal of its C-terminal autoinhibitory domain (AID), enabling calcineurin A to mediate the dephosphorylation of Drp1. The dephosphorylated Drp1 then transfers to mitochondria and mediates mitofission; (**4**) leads to ERK5 breakdown and subsequent downregulation of PGC-1α, decreasing gene expression related to mitochondrial biogenesis.

Increases in calpain expression and/or activity in the cytoplasm have been observed in cardiac injuries related to myocardial infarction [74], ischemia/reperfusion [29], diabetes [26], viral myocarditis [75] and drug-induced myocardial impairment [45]. It is known that the activation of cyto-calpain plays a role in the degradation of myocardial structural proteins and impairs cardiac dysfunction [65,76]. However, cyto-calpain may also impose detrimental effects on mitochondria, either by influencing participants involved in mitochondrial quality control (mitophagy and fusion/fission) and the mitochondrial apoptotic pathway or by modulating the transcription of genes related to mitochondrial biogenesis (Figure 1).

#### 3.1.1. Impairment of Mitochondrial Quality Control

Mitophagy, which refers to the selective degradation of mitochondria by autophagy [77], is an important mitochondrial quality control mechanism that helps to remove dysfunctional mitochondria in a timely fashion [78]. Several key modulators of mitophagy are calpain substrates [79,80]. Beclin 1 is an important autophagy effector required for nucleation of the phagophore and omegasome formation [81]. Following mitophagic stimuli, Beclin 1 relocalizes to the contact sites between the endoplasmic reticulum (ER) and mitochondria called mitochondria-associated membranes (MAM), where it interacts with PTEN-induced putative protein kinase 1 (PINK1) to promote the formation of autophagosome precursors [82]. Calpain-dependent Beclin 1 cleavage and autophagy defects have been observed in pathological conditions such as retinal ischemic injury [79], liver ischemia/reperfusion injury [83], neuronal oxidative injury [84] and inflammatory colitis [85], suggesting the participation of calpain-mediated mitophagy impairment during oxidative stress and inflammation, two mechanisms constantly involved in cardiac diseases. In support of this notion, Chen Qun and colleagues demonstrated that during cardiac ischemia/reperfusion, activated cyto-calpain leads to the degradation of cytosolic Beclin 1 and decreases the content of microtubule-associated protein 1 light chain 3B (LC3B), which can be restored by the calpain inhibitor [10]. Furthermore, inhibition of calpain either by transgenic overexpression of calpastatin in vivo or calpain inhibitor in vitro benefits mitophagy during cardiac ischemia/reperfusion injury [86]. Thus, activation of cyto-calpain may participate in the process of mitochondrial dysfunction by preventing the removal of the damaged mitochondria through depletion of key components of the mitophagy process.

In addition to mitophagy, cyto-calpain impairs mitochondrial quality control by promoting mitochondrial fission. Dynamin-related protein-1 (Drp1) is a cytoplasmic GTPase that is critical for mitochondrial fission and mitophagy [87,88]. Under stressed conditions, such as cardiac lipid-overload and ischemia/reperfusion, Drp1 is activated and translocates to the mitochondrial outer membrane, where it promotes mitochondrial fission and subsequent cardiac dysfunction [89,90]. Drp1 translocation to the mitochondria is closely associated with cytosolic calcium overload [89] and calcineurin-mediated dephosphorylation [91]. Interestingly, calpain was reported to enhance calcineurin A activity through cleavage of its C-terminal autoinhibitory domain [92]. Thus, it is possible that cyto-calpain may mediate mitochondrial fission under stress. This is supported by a recent work from Guan et al. [86]. In ischemia/reperfusion injured mouse heart and hypoxia/reoxygenation treated cardiomyocytes, Drp1 translocates from cytoplasm to mitochondria, concurrent with deformities of mitochondrial morphology. Inhibition of calpain activity alleviates Drp1 accumulation to mitochondria and decreases mitochondrial fragmentation [86], suggesting that cyto-calpain contributes to cardiac ischemia/reperfusion injury by inducing excessive mitochondrial fission.

#### 3.1.2. Initiating Mitochondria-Dependent Apoptosis

Cyto-calpain imposes detrimental effects on mitochondrial integrity through modulating key components involved in the mitochondrial apoptotic pathway. Studies have shown that many members of the B-cell lymphoma 2 (Bcl-2) family are calpain substrates. The Bcl-2 family, which comprises anti-apoptotic members and pro-apoptotic members, plays critical roles in mitochondria-dependent apoptosis [93]. The pro-apoptotic members Bcl-2-associated X protein (Bax) and BH3 interacting domain death agonist (Bid) normally reside in the cytosol of undisturbed cells [93]. Upon cleavage by calpain, truncated Bax translocates to the mitochondrial outer membrane and elicits apoptosis, which cannot be prevented by Bcl-2 [94,95]. Bid is a classic substrate for calpain [45,96]. At the onset of apoptosis, Bid is truncated to its active form tBid, which transfers to the mitochondrial outer membrane where it forms oligomers with Bax. The oligomers of Bax and tBid reorganize the mitochondrial outer membrane to form pores and promote the release of mitochondrial contents [93]. For example, in isolated rabbit hearts during ischemia/reperfusion, activated calpain cleaves Bid to generate tBid, leading to cytochrome C release and apoptosis [96]. In an in vitro model of cardiac hypertrophy, knockdown of calpain-1 by its specific siRNA significantly decreases the levels of tBid and cytochrome C release [46]. These studies have provided promising evidence to support the engagement of calpain activation in mitochondrial apoptosis through the cleavage of Bid and/or Bax.

#### 3.1.3. Indirectly Influencing Mitochondrial Biogenesis

Mitochondrial biogenesis refers to the growth and replication of pre-existing mitochondria [97], a process accomplished under the coordination of both the nuclear and mitochondrial genomes. Peroxisome proliferator-activated receptor γ (PPARγ) coactivator-1α (PGC-1α) is a powerful coactivator of transcription factors including PPARγ and nuclear respiratory factors (NRFs) [98], which regulate the transcription of numerous nuclear-encoded mitochondrial genes [99]. PGC-1α is a key driver of mitochondrial biogenesis in the heart [100] and plays a pivotal role in cardiac metabolism [101]. Amino acid sequence analysis shows that PGC-1α contains several domains enriched in proline (P), glutamic acid (E), serine (S) and threonine (T) (PEST domain) [102,103], a putative intramolecular signal for rapid proteolytic degradation by multiple proteases, including calpains [104,105]. Cell-free degradation assay showed that purified calpain 1 is capable of degrading PGC-1α [103], identifying PGC-1α as a direct substrate of calpain 1. Although the ubiquitin-proteasome pathway is responsible for basal PGC-1α turnover [102,103], calpain seems to be the executor of oxidant- and Ca^2+^-mediated PGC-1α degradation, since oxidant exposure and Ca^2+^ overload induce PGC-1α degradation that is sensitive to the calpain inhibitor instead of proteasome inhibitor [103]. This indeed suggests a possibility that under conditions of oxidative stress and/or calcium overload, two well-established mechanisms contributing to cardiac diseases, calpain dampens mitochondrial biogenesis through direct degradation of PGC-1α. Moreover, the work from Wei Liu and colleagues supported the detrimental role of calpain on the upstream of PGC-1α pathway [106]. In metabolic stress-related cardiomyopathy, excessive ROS production from gp91*^phox^* leads to the activation of calpain-1 and subsequent breakdown of ERK5. Loss of ERK5 and its resultant downregulation of PGC-1α decreases expression of genes related to mitochondrial biogenesis and oxidative phosphorylation (OXPHOS), thus imposing detrimental effects on mitochondrial functions, as evidenced by mitochondrial DNA depletion and impairment of mitochondrial morphology and OXPHOS activity. Prevention of ERK5 loss by scavenging cytosolic ROS or by blocking calpain-1 rescues mitochondrial function [106], indicating that cyto-calpain may mediate mitochondrial damage indirectly by modulating expressions of genes related to mitochondrial biogenesis and OXPHOS.

#### 3.1.4. Others

Under hypoxic conditions, HIF-1α is upregulated. HIF-1α accumulates and is translocated to the nucleus, promoting expression of target genes including nuclear factor erythroid 2-related factor 2 (Nrf2) [107] and mitochondria-specific gene frataxin [108]. Nrf2 was reported to positively regulate mitochondrial function and biogenesis [109] while frataxin reduced mitochondrial iron load [108]. These previous findings indicate that HIF-1α signaling pathway may be an important mechanism protecting mitochondrial function. Interestingly, inhibition of calpain attenuated HIF-1α degradation under hypoxic conditions [110]. Thus, it is highly possible that calpain-mediated cleavage of HIF-1α may damage mitochondrial function in the heart under pathological conditions, particularly in ischemia/reperfusion injury where calpain is activated.

### 3.2. Mito-Calpain-Mediated Mitochondrial Damage

**Figure 2:**
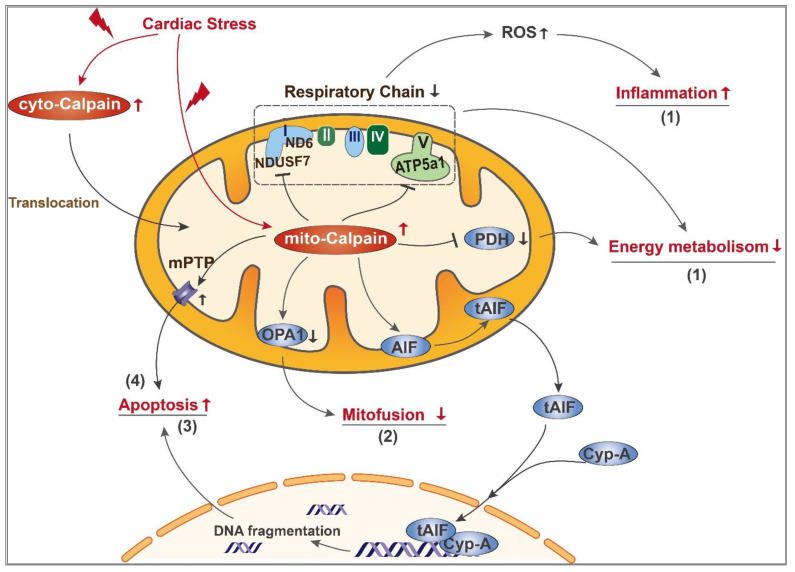
Schematic diagram of mito-calpain mediated mitochondrial damage in the diseased heart. In the diseased heart, cardiac stress leads to activation of both cyto-calpain and mito-calpain. Cyto-calpain can further translocate into mitochondria. Activated mito-calpain (**1**) cleaves subunits of complex I and complex V, leading to impairment of mitochondrial respiratory chain. On one hand, the resultant overproduction of ROS serves as a signaling molecule triggering the proinflammatory pathway; on the other hand, mitochondrial respiratory chain defects directly lead to ATP depletion. Moreover, mito-calpain degrades PDH, a critical enzyme in the tricarboxylic acid cycle, further dampening mitochondrial energy metabolism; (**2**) cleaves OPA1 and leads to decreased mitofussion and imbalance of mitochondrial dynamics; (**3**) mediates cleavage of AIF and its release from mitochondria to cytoplasm, where it interacts with cyclophilin A and further transfers to the nucleus. After entering the nucleus, tAIF binds to genomic DNA and promotes DNA fragmentation; (**4**) sensitizes mPTP opening, leading to mitochondrial swelling and loss of mitochondrial membrane potential, which further contributes to apoptosis.

In response to pathological conditions including ischemia/reperfusion (or hypoxia/reoxygenation), diabetes and sepsis, the protein levels and activity of calpain-1 and/or calpain-2 have been reported to increase in the mitochondria of cardiomyocytes and heart tissues [36,37,42]. Notably, increased calpain in mitochondria is closely associated with mitochondrial injury during ischemia/reperfusion [68,69], heart failure [40,111], sepsis [37] and diabetic cardiomyopathy [36], suggesting a potential role of mito-calpain in mediating cardiac pathology. This is indeed supported by our recent study, which demonstrated that transgenic up-expression of mitochondria-targeted calpain-1 sufficiently induced mitochondrial dysfunction, mitochondrial ROS generation, cardiomyocyte death and dilated heart failure in mice, leading to early death [40]. Studies have identified several mitochondrial proteins as substrates of mito-calpain [36,73,86,112]. Of note, alterations of these mitochondrial proteins may compromise mitochondrial energy metabolism, mitochondrial morphology and cell survival as these proteins are important for mitochondrial electron transport chain (ETC), mitochondrial dynamics and mitochondria-dependent apoptosis (see details in Figure 2 and below).

#### 3.2.1. Impairment of Mitochondrial Energy Metabolism

Activation of mito-calpain contributes to the damage of the ETC. Complex I is the first component of the ETC and resides in the mitochondrial inner membrane and transfers electrons from reduced nicotinamide adenine dinucleotide (NADH) to ubiquinone. Complex I comprises 44 different subunits, among which 14 are recognized as “core” subunits and the remaining 30 as “supernumerary” subunits [113]. Several subunits of complex I have been identified as mito-calpain targets, and mito-calpain-mediated cleavage of these subunits was reported to induce a dysfunctional ETC. During myocardial ischemia/reperfusion, activation of mito-calpain results in the degradation of the NADH:ubiquinone oxidoreductase core subunit S7 (NDUFS7), a subunit essential for complex I activity, which could be prevented by the calpain inhibitor MDL-28170 [10]. A recent study using isolated cardiac mitochondria showed that exogenous calcium treatment induces NDUFS7 degradation in mitochondria from wild-type mice but not *Capns1* knockout mice [114], providing direct evidence of mito-calpain-mediated NDUFS7 cleavage in the heart. Moreover, increased mito-calpain-2 is responsible for subunit ND6 cleavage and subsequent complex I dysfunction, leading to the opening of the mitochondrial permeability transition pore and myocardial injury following ischemia/reperfusion [69].

Our previous work identified ATP5A1 as a direct target of mito-calpain in the heart [36,37]. ATP5A1 is the F1 subunit of ATP synthase (complex V) which is crucial for ATP production. In type 1 diabetic mouse heart and cardiomyocytes cultured under diabetic conditions, mito-calpain-1 binds to and cleaves ATP5A1, leading to impairment of ATP synthase function, depletion of ATP production and excessive ROS production [36]. Similarly, in endotoxemic mouse hearts, we demonstrated that levels of calpain-1 protein and activity are increased in mitochondria, which colocalizes with and cleaves ATP5A1, leading to disruption of ATP synthase and oxidative stress [37]. A recent study further demonstrated that LPS stimulation dampens NAD-dependent deacetylase sirtuin-3 (SIRT3) activity in mouse heart, leading to an increase in ATP5A1 acetylation, which makes ATP5A1 more prone to degradation by calpain [115].

Pyruvate dehydrogenase (PDH) is a mitochondrial matrix enzyme that serves a critical role in mitochondrial energy metabolism by connecting glycolysis and tricarboxylic acid (TCA) cycle [116]. Under conditions of cardiac ischemia/reperfusion injury and endoplasmic reticulum stress, activation of mito-calpain leads to the degradation of PDH α_1_ subunit (PDHα_1_) in cardiac mitochondria [114,117], providing further evidence in support of mito-calpain-mediated disturbance in energy metabolism. Overall, activation of mito-calpain represents a new mechanism contributing to the impairment of mitochondrial energy metabolism in hearts under certain pathological conditions. Elucidating the roles of mito-calpain in modulating mitochondrial energy metabolism requires further investigations, including identification of new substrates of mito-calpain related to mitochondrial energy metabolism in diseased hearts.

#### 3.2.2. Imbalance of Mitochondrial Fission and Fusion

Mitochondria are highly dynamic organelles that undergo constant cycles of fusion and fission to maintain mitochondrial integrity and functions [118]. Mitochondrial fusion is the union of two adjacent mitochondria and serves as an essential complementation for damaged mitochondria to mitigate stresses by exchanging components with healthy mitochondria [119]. Mitochondrial fission is the division of one mitochondrion into two smaller mitochondria and favors mitophagy to sequester and eliminate dysfunctional mitochondria [119]. Fine-tuned mitochondrial fusion and fission is indispensable for cardiac homeostasis, since defects in either fusion or fission elicit cardiac dysfunction [88,120]. Dysregulated fusion and fission, often manifested as excessive fission and insufficient fusion, are constantly implicated in cardiac diseases [89,121,122]. In addition to mitochondrial fission promoted by the cyto-calpain/calcineurin A/Drp1 activation pathway (discussed above), mitochondrial deformation also occurs concurrently with an increase in mito-calpain activity in cardiac diseases, suggesting a potential role of mito-calpain in mediating impairment of mitochondrial morphology.

Mitochondrial fusion relies on the orchestration of three mitochondria-associated GTPase proteins, namely mitofusions 1 and 2 (MFN1 and MFN2), and optic atrophy type 1 (OPA1). MFN1 and MFN2 mediate the fusion of mitochondrial outer membrane [123]. MFN2 has been recognized as a direct substrate of calpain since in vitro calpain cleavage assays using recombinant proteins observed the cleavage of MFN2 by calpain in a dose- and time-dependent manner [124], although future work is needed to clarify whether this calpain-dependent cleavage of MFN2 contributes to cardiac diseases. OPA1 is dynamin-related GTPase protein located in the mitochondrial inner membrane and mediates the fusion of the mitochondrial inner membrane [122]. Besides, OPA1 has been shown to regulate mitochondrial cristae structure [125], suggesting a crucial role of OPA1 in maintaining mitochondrial morphology. A recent study pinpointed a key role of OPA1 in respiratory supercomplex assembly as OPA1 knockdown impairs the assembly and activity of ETC and damages OXPHOS [126]. OPA1 exists in two forms, as a full-length form (long form, L-OPA1) anchored to the mitochondrial inner membrane or a short form (S-OPA1) that is soluble within the cristae lumen space [125]. L-OPA1 is the prerequisite for mitochondrial fusion, whereas S-OPA1 accumulation is related to fission [122]. Perturbed OPA1 processing was reported in stress-induced cardiomyocytes and exerted deleterious effects on cardiac function [122]. OPA1 perturbation may result from calpain activation, since inhibition of calpain activity with overexpression of calpastatin restored mitochondrial morphology in an OPA1-dependent manner [127]. In a mouse model of myocardial ischemia/reperfusion injury, up-regulation of mitochondrial calcium unidirectional transporter (MCU) was reported to cause mitochondrial Ca^2+^ overload and subsequent calpain activation, which was accompanied by a dramatic decrease in OPA1 level and imbalanced mitochondrial dynamics [86]. Importantly, inhibition of calpain activity restored OPA1 protein level in ischemia/reperfusion-induced cardiomyocytes, while OPA1-knockout abolished the protective effect of calpain inhibition on mitochondrial dynamics [86]. Thus, OPA1 perturbation may be another downstream mechanism of calpain-activation-mediated ischemia/reperfusion injury. However, whether calpain directly targets OPA1 or modulates OPA1 expression through an indirect mechanism remains elusive.

#### 3.2.3. Mitochondrial Apoptosis

Mito-calpain contributes to caspase-independent apoptosis by mediating the translocation of apoptosis-inducing factor (AIF) from mitochondria to the cytosol. AIF is a mitochondrial oxidoreductase localized to the mitochondrial intermembrane space, where it binds to the mitochondrial inner membrane and helps to stabilize the respiratory chain [128]. Calpain-mediated cleavage of AIF allows its dissociation from the mitochondrial inner membrane and release from mitochondria [129]. The truncated AIF (tAIF) can interact with a variety of proteins in the cytosol, including hear shock protein 70 (HSP70) and cyclophilin A. HSP70 helps the tAIF remain in the cytoplasm [130], while cyclophilin A promotes its nuclear translocation [131]. After tAIF enters the nucleus, its C-terminus binds to genomic DNA and promotes their fragmentation during apoptosis [132]. Mito-calpain-mediated AIF truncation has been observed in the heart and contributes to cardiac injury [129,133]. Using purified mitochondria from mouse hearts, Qun Chen et al. demonstrated that exogenous calcium decreased AIF protein levels in a calpain inhibitor sensitive manner, suggesting a role of mito-calpain/AIF cascade in mediating cardiac injury [133].

Mitochondrial permeability transition pore (mPTP) opening leads to the loss of mitochondrial membrane potential and inhibition of ATP production, accompanied by the release of pro-apoptotic components and mitochondrial swelling [134]. In mitochondria isolated from rat hearts subjected to ischemia/reperfusion, Ca^2+^ overload concomitantly induced activation of mito-calpain-2, mPTP opening and mitochondrial swelling. Calpain inhibitor partially diminished mPTP opening elicited by Ca^2+^-overload, indicating that in addition to mito-calpain, additional mechanisms are operative in mediating mPTP opening in myocardial ischemia/reperfusion injury [69].

## 4. Precisely Targeted Inhibition of Mito-Calpain as a Potential Therapeutic Strategy for Cardiac Disease

Since calpain plays paramount roles in the pathogenesis of cardiac diseases, inhibition of calpain activity appears to be a promising strategy for treatment. A number of preclinical studies have demonstrated the therapeutic potential of several calpain inhibitors. For instance, in mammal models, inhibition of calpain activity with MDL-28170 or PD150606 provides protective effects in diabetic cardiomyopathy [135,136], sepsis-induced cardiomyopathy [115], and ischemia/reperfusion injury [72]. SNJ-1945, an oral calpain inhibitor, ameliorates mitochondrial dysfunction, reduces myocardial fibrosis and improves cardiac function in mice subjected to myocardial infarction [28]. In a rat model of myocardial ischemia/reperfusion, chronic oral administration of SNJ-1945 alleviates cardiac remodeling and dysfunction [137]. Apart from pharmacological inhibitors, genetic ablation of *Capns1* or transgenic overexpression of calpastatin protects the heart from various insults, including, but not limited to, diabetes [41], sepsis [37], lipotoxicity [39], and ischemia/reperfusion [54]. However, there has been no clinical trial using calpain inhibitors to treat cardiac diseases.

It is worthy of note that functional calpains are indispensable for cardiac homeostasis [20]. Calpain-1 was reported to promote ubiquitination and proteasomal degradation of a subset of myocardial proteins, thereby preventing aggregation of nondegraded proteins and subsequent cardiomyocyte dysfunction [19]. Calpain-2 knockout causes an embryonic lethal effect, highlighting a crucial role of calpain for embryonic development [138]. In addition, our previous work has demonstrated a protective role of calpain-2 against doxorubicin-induced cardiac injury [139] and heat-stress-induced cardiac dysfunction [140]. Thus, global inhibition of calpain within cardiomyocytes may not be the best strategy for cardiac protection. Given the active roles of mito-calpain in mediating mitochondrial injury in cardiac diseases, targeted inhibition of calpains in mitochondria provides a more appealing approach. Recently, using transgenic mice overexpressing mitochondrial-targeted calpastatin in cardiomyocytes, we demonstrated that selective inhibition of mito-calpain ameliorated oxidative stress, mitochondrial dysfunction and myocardial dysfunction elicited by ischemia/reperfusion and/or hyperglycemia [42]. The protective effects of mitochondrial-targeted calpastatin were attributed to preservation of ATP synthase activity and prevention of mitochondrial ROS production [42]. Further, targeted inhibition of mito-calpain may be a safe strategy for therapy as transgenic mice with over-expression of cardiomyocyte-specific and mitochondria-targeted calpastatin exhibited no abnormal cardiac phenotypes [42]. Taken together, these results indicate that specifically targeting calpain in mitochondria may be an alternative strategy for alleviating oxidative stress-related cardiac diseases. Further investigations are necessary to demonstrate the feasibility, safety and efficacy of targeted inhibition of mito-calpain in different pre-clinical models of cardiac diseases and heart failure. Ultimately, pharmaceutical inhibitors targeting mito-calpain may be considered for cardiac disease treatment.

## 5. Concluding Remarks

**Figure 3:**
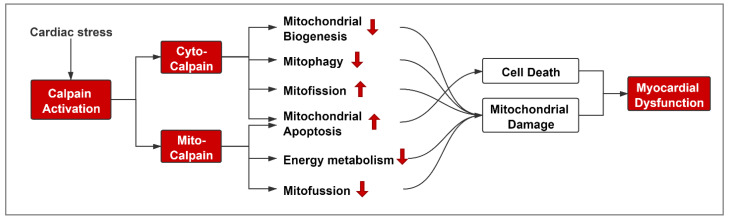
Calpain-mediated mitochondrial injury contributes to cardiac diseases. Under pathological conditions, cardiac stress leads to overactivation of calpain, including cyto-calpain and mito-calpain. Cyto-calpain contributes to impairment of mitochondrial biogenesis and mitophagy, promotes mitofission and mitochondrial apoptotic pathway. Mito-calpain dampens mitofussion and mitochondrial energy metabolism and facilitates mitochondrial apoptosis. Thus, cyto-calpain and mito-calpain work separately yet synergistically in mediating mitochondrial damage and cell death, leading to myocardial dysfunction and eventually contributing to the progression of heart failure.

Dysregulation of calpain contributes to the pathogenesis of cardiac diseases and progression of heart failure. This review demonstrates that different subcellular localized calpains may work synergistically to dampen mitochondrial function and promote cardiac injury and dysfunction (Figure 3). Thus, prevention of mitochondrial dysfunction from calpain-mediated damage may be a new therapeutic strategy for cardiac disease treatment. Indeed, selective inhibition of mitochondria-localized calpain through up-regulation of mitochondria-targeted calpastatin offers a feasible and precise resolution to alleviate oxidative-stress-related cardiac pathology in preclinical animal models [42]. However, up-regulation of mitochondria-targeted calpastatin is currently difficult to attain in clinical settings. Therefore, accurately targeting calpain activity in mitochondria using its pharmacologic inhibitors remains a challenge. Moreover, the availability of calpain inhibitors is currently very limited for clinical trials. Although a calpain inhibitor ABT-957 had been advanced to a phase I clinical trial (AbbVie, Phase I, terminated, Clinical Trials.gov, NCT02220738) [141] for the treatment of Alzheimer’s disease, the pharmacodynamic results were not supportive of continued development [142]. Recently, a novel calpain inhibitor BLD-2660 was approved for a phase II clinical trial (BLADE Therapeutics, Phase II, active, Clinical Trials.gov, NCT04334460) for the treatment of COVID-19 [143], which could show promise for further calpain inhibitor development. At present, the pharmaceutical development of calpain inhibitors represents a significant challenge in the field.

In the future, ongoing studies are needed to further elucidate the mechanisms underlying calpain-mediated mitochondrial dysfunction in the pathogenesis of cardiac diseases. Specifically, multidisciplinary collaborations are necessary to better identify direct targets of subcellular localized calpains, as well as develop therapeutic strategies that can precisely target mito-calpain for cardiac disease treatment.

## Data Availability

Not applicable.
